# Learning from nature: photosynthetic traits conferring superior salt tolerance in wild rice *Oryza coarctata*

**DOI:** 10.1098/rstb.2024.0242

**Published:** 2025-05-29

**Authors:** Ping Yun, Babar Shahzad, Md Hasanuzzaman, Tahmina Islam, Lana Shabala, Meixue Zhou, Gayatri Venkataraman, Zhong-Hua Chen, Sergey Shabala

**Affiliations:** ^1^ The University of Western Australia, Perth, Australia; ^2^ University of Tasmania, Hobart, Tasmania, Australia; ^3^ Sher-e-Bangla Agricultural University, Dhaka, Dhaka District, Bangladesh; ^4^ Department of Botany, University of Dhaka, Dhaka, Bangladesh; ^5^ MS Swaminathan Research Foundation, Chennai, Tamil Nadu, India; ^6^ School of Agriculture, Food and Wine, Waite Research Institute, University of Adelaide, Adelaide, Australia

**Keywords:** salinity, wild rice, stomata, ABA, photosynthesis, gas exchange

## Abstract

This work aimed to reveal a mechanistic basis of differential salinity stress tolerance between cultivated (*Oryza sativa*) and wild (*Oryza coarctata*) rice species related to photosynthesis and leaf gas exchange. With an innate larger (twofold) stomata, *O. coarctata* could achieve a similar net photosynthetic rate with 63% lower stomatal density and show 72% higher intrinsic water use efficiency under control conditions. After salt treatment, cultivated rice developed smaller and denser stomata with decreased aperture, which resulted in lower stomatal conductance and reduced stomatal opening speed. Also, *ca* twofold decrease in the maximum carboxylation rate of ribulose bisphosphate carboxylase (RuBisCO) and the rate of ribulose 1,5-bisphosphate (RuBP) regeneration were also observed. In addition, Na^+^ accumulation in mesophyll depressed the operation of photosystem II (PSII). At the same time, *O. coarctata* maintained relatively steady CO_2_ assimilation under saline conditions, with both stomatal and photochemical traits largely unaffected. This included unaltered stomatal size, density and opening speed; enhanced ribulose bisphosphate carboxylase carboxylation and RuBP regeneration in the Calvin cycle; and more stable leaf photochemistry. *Oryza coarctata* also possessed an ability to utilize Na^+^ for stomata operation—a trait lacking in cultivated rice. The above-mentioned traits could be used as potential targets in breeding programmes to improve salinity tolerance in cultivated rice.

This article is part of the theme issue ‘Crops under stress: can we mitigate the impacts of climate change on agriculture and launch the ‘Resilience Revolution’?’.

## 1. Introduction

Soil salinization is one of the biggest global environmental threats that hinder crop production, with more than 1257 million hectares being salt-affected [1]. Most crops are glycophytes and, hence, strongly affected by soil salinity. As a major staple cereal that feeds half the population across the world, rice (*Oryza sativa*) has a much lower salt-tolerance threshold compared to other monocotyledonous crops (e.g. barley and wheat), unable to survive salinities exceeding 100 mM NaCl (20% of seawater level) [2]. One of the reasons for this is detrimental effects of soil salinity on stomata operation and CO_2_ assimilation in rice leaves. Plant biomass (hence, grain yield) is ultimately proportional to the amount of CO_2_ assimilated during their ontogeny, and salt stress-induced reduction in stomata conductance (Gs; termed as stomatal limitation) and toxic effects of accumulated Na^+^ to leaf photochemistry (non-stomatal limitation of photosynthesis [3]) result in major decline in net CO_2_ assimilation.

Stomata are the pores in the leaf surface that allow leaf gas exchange and transpirational water loss. Stomatal conductance reflects gas diffusion and has several determinants, including stomatal size, density and aperture. In the stomatal limitation of photosynthesis, both stomatal development and response speed are key elements. The intrinsic stomatal patterning varies among different species with respect to density, size, aperture area and Gs [4], this is specifically true for *Oryza* species where the natural variation of stomatal conductance correlated positively with stomatal density (SD) [5]. At the same time, SD is strongly affected by salinity stress [6]. In halophytes (naturally salt-loving plants) and some salt-tolerant glycophytes (such as barley), SD is reduced under saline conditions [6–9], while in salt-sensitive cereal crops (such as wheat), SD is increased [10]. The above-mentioned reduction in SD is believed to be a developmental response [6] and is attributed as an attempt to reduce water loss by residual transpiration. Surprisingly, the data reporting effects of salinity stress on SD in rice is scarce. Would reduction in SD benefit its water use efficiency (WUE)? Or is extremely high salt sensitivity in rice related to specific Na^+^ toxicity and will be unaffected by any changes in SD? The only possible hint came from that study using transgenic rice with decreased SD (about 60% of its wild type) that was shown to possess better drought tolerance and higher WUE [11]. Also, this study showed that plants treated with 20 mM NaCl performed better than wild type [11]. However, as 20 mM NaCl is below the salt threshold (4 dS m^−1^, equivalent to 40 mM NaCl) [2], the question of whether reduced SD would contribute to salt tolerance in rice remains to be answered.

Another factor contributing to stomatal limitation of photosynthesis is stomata speed, with the notion that speedy stomata operation will minimize the antagonism between water conservation and CO_2_ supply [12] and, hence, improve WUE. Faster stomatal responses enable plants to efficiently cope with variable environmental stresses under field conditions, which require frequent adjustment of stomatal movement, considering the temporal asynchrony between stomatal kinetics and photosynthetic rate dynamics under fluctuating conditions [9,13]. The rapidity of Gs response is determined by anatomical, structural and biochemical features [14]. It was shown that halophytic quinoa and sea beet species had faster stomatal opening speeds than their glycophytic relatives under both control and salt conditions [9]. No such comparison has been done for rice and its halophytic relatives. The operation (opening and closure) of stomata is achieved by adjusting the turgor of its guard cells (GCs), which rely on ionic osmolytes or organic compounds [15,16] and is modulated by abscisic acid (ABA). The ABA signalling pathway is highly conserved across land plants [17] but is strongly affected by abiotic stress conditions [18,19]. Under salinity conditions, ABA levels increase in leaf tissues due to osmotic stress, which consequently induces stomatal closure to prevent water loss [20]. Interestingly, both endogenous ABA levels and the extent of their increase are much smaller in halophytes compared with glycophytic species [15]. It was shown that exogenously supplying ABA could alleviate salt-induced photodamage and induce salt tolerance in rice [21,22], but the causal relationships between salinity stress, ABA signalling and stomatal operation in cultivated and wild rice relatives have never been studied, to the best of our knowledge.

Non-stomatal factors that restrict photosynthesis are primarily photochemical and biochemical processes in chloroplasts. Several constraints are involved in these reactions such as the amount of chlorophyll, the electron transfer rate between photosystems (PS) and the activity of photosynthesis-related enzymes in Calvin cycle (e.g. ribulose bisphosphate carboxylase (RuBisCO)) and gluconeogenesis (e.g. fructose 1,6-bisphosphatase (FBPase) and fructose-bisphosphate aldolase (FBP aldolase)) processes [3,20]. Salt stress has a major impact on chloroplasts (ultrastructure and function) due to hindered ion homeostasis, leading to accumulation of reactive oxygen species (ROS) [23]. Ion transport between cytosol and stroma is essential for optimal chloroplast functions as the chloroplastic membrane contains many transporters and proteins [23]. Under saline conditions, ion imbalance (e.g. excessive Na^+^ and Cl^−^ accumulation, K^+^ loss) occurred in mesophyll cells, consequently causing chloroplast thylakoid swelling, grana unstacking, protein inactivation and chlorophyll degradation [24,25]. At the same time, salt-induced K^+^ loss would impair photosynthetic processes, as K^+^ is essential for both chloroplast ultrastructure and RuBisCO activity [26]. Na^+^ accumulation also causes depolarization of plasma membrane, therefore affecting proton motive force across membranes [27]. This may interfere with anion transport through voltage-dependent anion channels in chloroplasts [23]. Although Cl^−^ is an essential element for plants, the salt-induced Cl^−^ proliferation could hinder the functional activity of PSII oxygen-evolving Mn–Ca–Cl complex, due to the existence of high-affinity Cl^−^ binding sites, and lead to photoinhibition and elevated ROS production [24]. The effects of salt stress on the PS operation and electron transfer in *O. sativa* include inactivation of PS reaction centre and an increase in energy dissipation [25]. Also, the relative electron transport rate from PSII to photosystem I (PSI) of the chloroplast isolated from glycophytes is inhibited by NaCl [28]. Such suppression could be attributed to inhibition of photosynthetic protein synthesis (such as PSII D1 protein (PsbA) and PSII extrinsic protein O (PsbO)) and reduction in ATP synthesis for repair of PSII complex [3,29]. Salt-induced osmotic stress inactivates oxygen evolution in PSI and PSII complexes in a rapid manner [30], and biosynthesis of osmoprotectants such as proline and inositol is able to stabilize PS activity and increase photosynthetic pigments under saline conditions [21,22,31,32].

*Oryza coarctata* (also known as *Porteresia coarctata*) is the only halophytic species in the *Oryza* genus that is mainly found around the coast area of India, Pakistan and Bangladesh [33]. *Oryza coarctata* is an allotetraploid wild rice (with KKLL genome) that can withstand seawater submerging twice a day [34]. It possesses a unique anatomical structure compared to cultivated rice, such as a C_3_–C_4_ intermediate (Kranz-like anatomy) thick leaf structure, a thick cuticle layer on the leaf surface to conserve water and intrinsic larger and sparser stomata [5,35]. Previous studies showed that as a halophyte, *O. coarctata* also has distinguished salt-tolerant enzymes for photosynthesis protection. For instance, the *in vitro* activity of chloroplastic fructose-1,6-bisphosphatase (chloroplastic FBPase) from *O. coarctata* performs better than *O. sativa* in response to salt treatment [36]. However, despite the fact that *O. coarctata* has been identified as a salt-tolerant species for decades, comprehensive comparative analyses of leaf gas exchange and primarily photochemical traits between salt-grown *O. coarctata* and its cultivated *O. sativa* counterparts have never been conducted. This work aimed to fill in this gap. Specific questions to be answered were (i) what is the role of changes in stomata patterning as a determinant of salinity stress tolerance between cultivated and wild rice species? (ii) Are the changes in stomata patterning due to a switch in the developmental programme, or determined by differences in the cell size? (iii) How does the leaf photochemistry in halophytic species *O. coarctata* respond to saline conditions? (iv) How do *O. coarctata* leaves deal with accumulated salt? (v) Which component of photosynthetic limitation—stomatal or non-stomatal—makes the biggest impact on plant performance under saline conditions?

To answer the above-mentioned questions, we have conducted a comparative analysis of leaf gas exchange characteristics, stomatal patterning and leaf photochemical traits between salt-sensitive cultivated rice (*O. sativa*, cv. Koshihikari) and wild species *O. coarctata*, focusing on the stomatal and non-stomatal limitations of photosynthesis in both species. The overarching aim of this study was to identify the potential of incorporating photosynthetic-related traits from *O. coarctata* into cultivated *O. sativa* species through breeding programmes, to reduce salt stress-induced yield losses in cultivated rice.

## 2. Material and methods

### (a) Growth conditions

*Oryza sativa* (subsp. *japonica*, *cv*. Koshihikari, seeds donated by SunRice) and its halophytic species *O. coarctata* (seedlings obtained from MS Swaminathan Research Foundation, India) were used a s plant materials. Germinated *O. sativa* seeds were sown into a standard potting mix [37]. After seedlings emerged, uniformed plants were transplanted into 1.5 l pots (3–4 seedlings per pot), which contained a mixture (25/75% v/v) of clay soil and standard potting mix [38]. For *O. coarctata*, seedlings were propagated in the soil mixture (described above), and newly developed seedlings were separated from the clumps and transplanted into pots. Pots with seedlings were put in 15 l water-filled tubs and grown until the four-leaf stage for the following experiments.

For experiments with *intact plants*, plants were treated for four weeks with salinity (100 mM NaCl solution). The NaCl concentration was gradually increased from 25 to 100 mM in the first 3 days, and solutions were replaced every 3 days. Leaf gas exchange and stomatal characteristics were measured at the end of treatment. In addition, experiments with *excised leaves* and *floating leaf segments* were conducted. The second youngest leaf was excised from control plants and then treated with various solutions (details described below).

### (b) Leaf gas exchange and stomatal operation characteristics

The gas exchange characteristics such as net photosynthetic rate (Pn), stomatal conductance (Gs), intercellular CO_2_ concentration (Ci) and transpiration rate (Tr) were measured by a portable gas exchange system (Li-6400XT, Li-Cor, USA) from plants treated with NaCl for one month. Measurements were performed between 10.00 and 15.00, under the conditions of 1500 μmol m^−2^ s^−1^ light density, 400 μmol mol^−1^ CO_2_ concentration, 400 μmol s^−1^ flow rate, relative humidity 65–70% and 25℃ chamber temperature. Leaves were equilibrated under the described conditions (*ca* 5−10 min) before readings were recorded.

The CO_2_ curve measurements were conducted under settings of 1500 μmol m^−2^ s^−1^ photosynthetic photon flux density (PPFD) and 1.1 kPa vapor pressure deficit (VPD). Leaves were firstly equilibrated in the chamber under 400 ppm CO_2_, followed by 10 levels of CO_2_ concentration (50, 100, 200, 300, 400, 500, 700, 900, 1200 and 1500 μmol mol^−1^). Parameters were recorded after the CO_2_ exchange had reached a steady state. The R package ‘Plantecophys’ was used to calculate the maximum carboxylation rate of RuBisCO (*V*_cmax_) and rate of RuBP regeneration via electron transport (*J*_max_) [39].

The rapid light curve (RLC) measurements were performed by the Auto program in the Li-6400XT. Stabilized Pn was recorded under nine light intensities (from 2000 to 0 μmol m^−2^ s^−1^) accompanied by 400 μmol mol^−1^ CO_2_ concentration and 400 μmol s^−1^ flow rate. Variables derived from *A/I* (*Pn*/*I*) curve were calculated by the non-rectangular hyperbola-based model of an Excel tool proposed by de Lobo *et al.* [40, eqn (6)]. Although Pn from the RLC was lower compared with the CO_2_ responses curves (due to batch differences) the effect of salt on the relative Pn responses (% of control) was the same.

**Figure 1.**
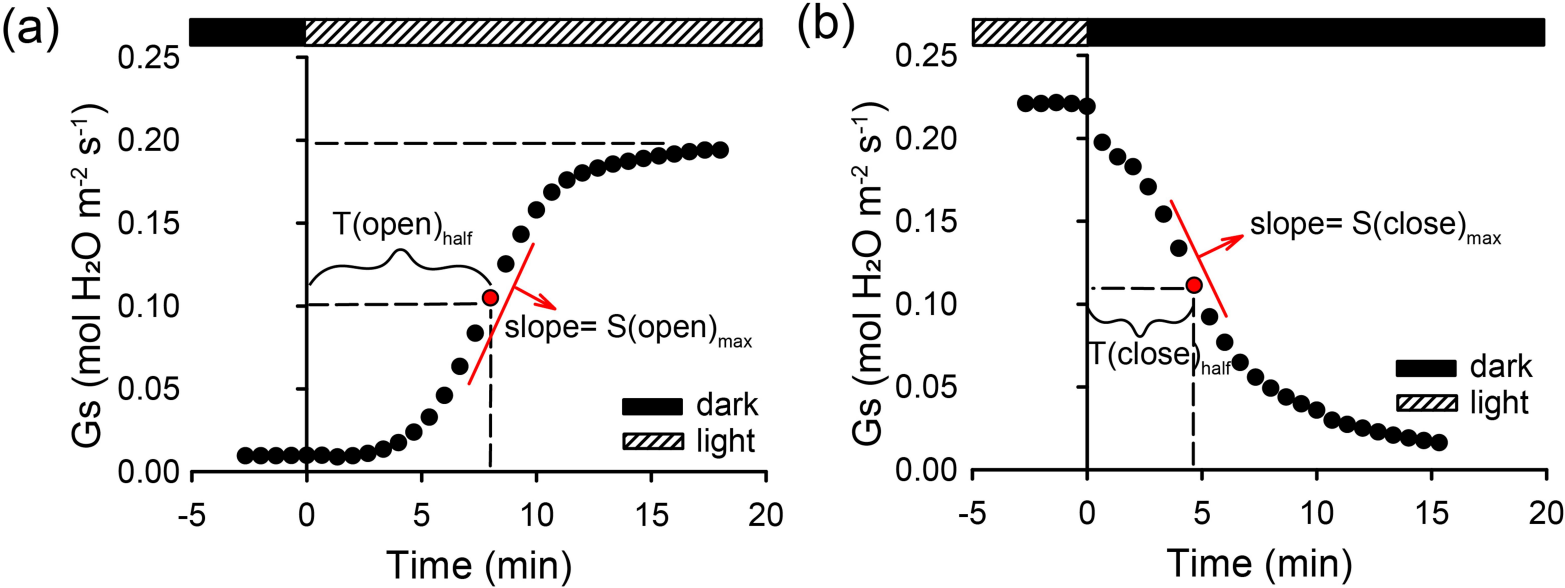
(a,b) Schematic figures show calculation methods for parameters derived from dynamic stomatal response to light/dark stimulation. Plants were pre-adapted to dark conditions for 2 h under 400 μmol mol^−1^ CO_2_ concentration and 400 μmol s^−1^ flow rate. After recording their Gs values for 5 min, plants were exposed to a bright (1500 μmol m^−2^ s^−1^) light, and Gs was measured every 30 s until it reached saturation and was stable. The light was then turned off, and the kinetics of Gs response to darkness was measured. Stomatal conductance was recorded every 30 s from intact seedlings treated with 0 or 100 mM NaCl for one month. *T*_half_ is the response time taken for Gs to reach half of the maximum value, and the *S*_max_ is the slope of the linear part of the Gs response curve.

To evaluate the speed and kinetics of stomatal movements, plants were pre-adapted to dark conditions for 2 h under 400 μmol mol^−1^ CO_2_ concentration and 400 μmol s^−1^ flow rate. After recording Gs values for 5 min, plants were exposed to a bright (1500 μmol m^−2^ s^−1^) light, and Gs was measured every 30 s until it reached saturation and was stable. The light was turned off then, and the kinetics of Gs response to darkness was measured. Maximum stomatal opening or closure speed (∆Gs min^−1^) was estimated as a slope of the linear part of the Gs versus time curve (figure 1).

### (c) Stomatal aperture length, density and index

The epidermal imprints method was used to quantify stomatal geometry, density and index. On the abaxial surface, the middle part of the youngest fully expanded leaf (developed during salt treatment) was coated with a thin layer of clear nail polish. After drying, the nail polish layer was gently removed with transparent tape and attached to a glass slide, then a photo was taken. The number of stomata and epidermal cells in the field of view was counted to calculate the SD and stomatal index (SI). The SI was represented as the ratio between stomatal numbers and the total number of stomata and epidermal cells in the same field of view. The stomatal size was determined by stomatal aperture length (SAL) [5], which was measured by Image J software (NIH, USA).

### (d) Experiments with excised leaves

Experiments with excised leaves were conducted to eliminate the impact of the possible difference in root-related traits between two species on Na^+^ uptake and delivery to the shoot. An excised leaf was immobilized in an upright position by the spongy foam in 50 ml Falcon tubes, which contained 40 ml of 1/8 Hoagland solution plus appropriate treatments (NaCl, ABA, Mannitol; electronic supplementary material, figure S1; see also Wu *et al*. [41] for more details). Tubes were then placed in the same growth chamber, which was used for growing seedlings. For ABA treatment, tubes were covered with aluminium foil paper to prevent direct exposure to light, and the solution was refreshed daily.

The maximum potential quantum efficiency of PSII (Fv/Fm) was measured from leaves dark-adapted for 30 min. Gs was measured by leaf porometer (SC-1, ICT International, Australia) between 10.00 and 15.00. The transpiration rate (ml cm^−2^) was calculated as the amount of solution evaporated from the tube (in ml) divided by the total leaf area (cm^2^) of plants in the same tube.

### (e) Floating leaf disc experiments

As the difference in transpiration rate between *O. sativa* and *O. coarctata* could potentially result in a different Na^+^ loading in leaf lamina, floating leaf disc experiments were conducted, where NaCl treatment was applied directly to leaf mesophyll through the cut edge. To do this, the middle part of the excised leaf was cut into 1 cm long segments and floated in Petri dishes that contained various concentrations of NaCl and KCl solution.

### (f) Leaf K^+^ and Na^+^ ion content

Treated excised leaves were washed with running tap water to remove residual nutrient solution and dried in an oven at 80 ℃ until they reached a constant weight. The dried leaf samples were then cut into small pieces and transferred to glass test tubes for the ddH_2_O digestion method [42] in a boiling water bath for 2 h. After filtering through filter paper, the liquid extraction was diluted to measure the ion content of K^+^ and Na^+^ by flame photometer (Jenway PFP7/C, Cole-Parmer, USA).

### (g) Statistical analysis

The SPSS software (v. 28.0, IBM, USA) was used for statistical analysis. All significance comparisons were determined by using either Student’s *t*‐test or ANOVA.

## 3. Results

### (a) Salinity effects on gas exchange, carbon dioxide and light response curve

**Figure 2.**
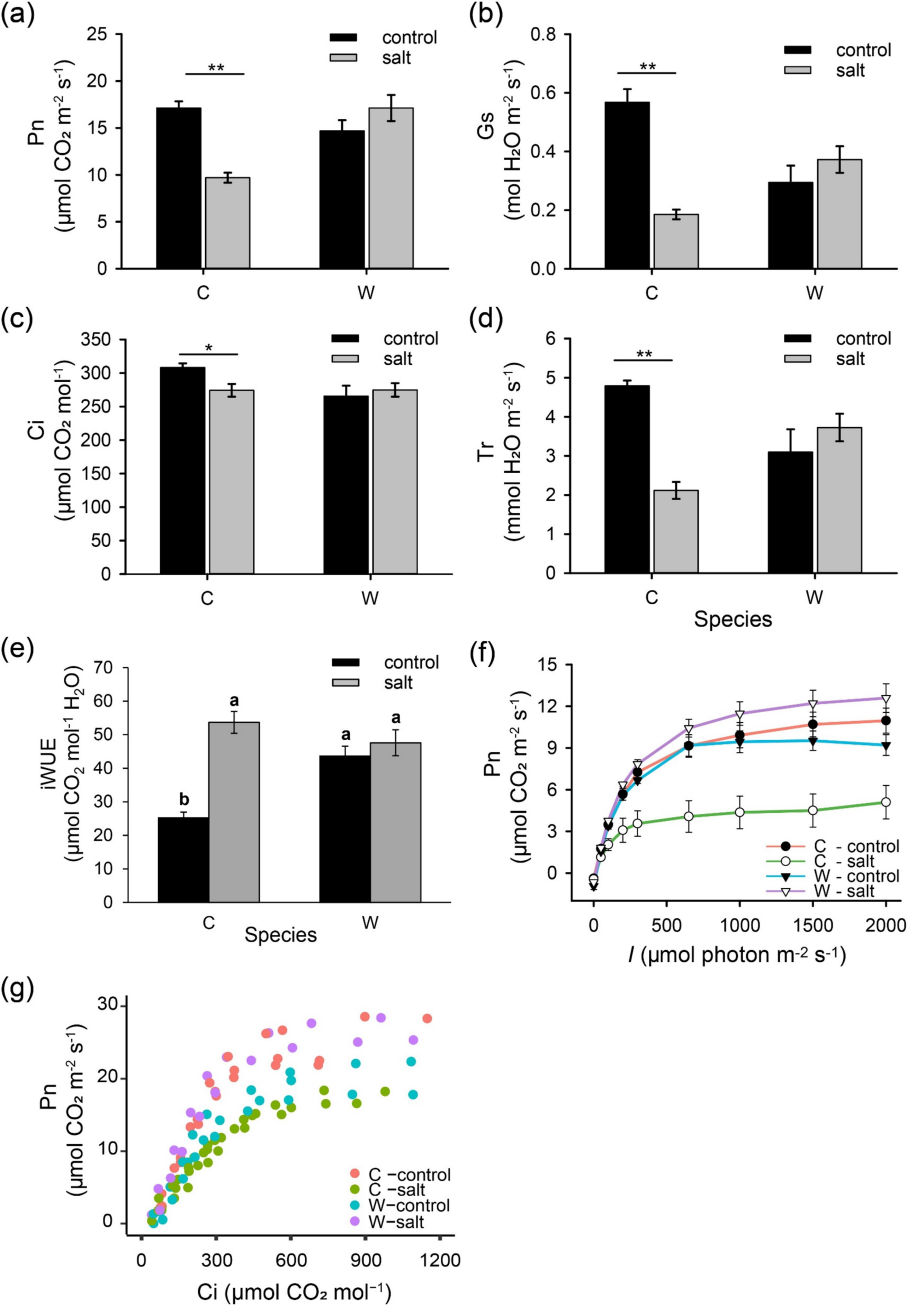
Alterations in leaf gas exchange and light and CO_2_ response curves from intact *O. sativa* and *O. coarctata* plants treated with 100 mM NaCl for one month. (a) Pn—net photosynthetic rate, (b) Gs—stomatal conductance, (c) Ci—intercellular CO_2_ concentration, (d) Tr—transpiration rate. Measurements were performed between 10.00 and 15.00, under the conditions of 1500 μmol m^−2^ s^−1^ light density, 400 μmol mol^−1^ CO_2_ concentration, 400 μmol s^−1^ flow rate, relative humidity 65–70% and 25°C chamber temperature. (e) Leaf iWUE of two species. iWUE is calculated as the net photosynthesis rate (Pn) divided by stomatal conductance to water vapour (Gs) [43]. (f,g) Data points for (f) light response curve (*A/I*) and (g) CO_2_ response curve (*A/Ci*), and variables calculated by models are shown in table 1. For *A/I* curve, stabilized Pn was recorded under nine light intensities (from 2000 to 0 μmol m^−2^ s^−1^) accompanied by 400 μmol mol^−1^ CO_2_ concentration and 400 μmol s^−1^ flow rate. For *A/Ci* curve, measurements were conducted at a setting of 1500 μmol m^−2^ s^−1^ PPFD and 1.1 kPa VPDL. A leaf was firstly equilibrated in the chamber under condition of 400 ppm CO_2_, followed by 10 levels of CO_2_ concentration (50, 100, 200, 300, 400, 500, 700, 900, 1200 and 1500 μmol mol^−1^). To enhance the visual distinction between treatments, adjacent data points were connected using coloured lines in (f). C—cultivated rice *O. sativa*, W—wild rice *O. coarctata*. Values are mean ± SE (*n* = 3–7). * and ** indicate significant differences between species with *p* < 0.05 and *p* < 0.01 by Student’s *t-*test, respectively. Different letters indicate significant differences with *p* < 0.05 by ANOVA.

Long-term salt stress has a significant negative impact on the overall growth of cultivated rice (electronic supplementary material, figure S2). At the same time, *O. coarctata* performance was stimulated by 100 mM NaCl treatment. The net photosynthetic rate, stomatal conductance and transpiration rate of cultivated rice dropped dramatically (by 43, 68 and 55%, respectively) after being treated with 100 mM NaCl for one month. This decline in Pn was mostly associated with Gs but not Ci (figure 2). On the contrary, in *O. coarctata*, Pn, Gs and Tr show a slight increase, and Ci was unaffected under saline conditions (figure 2). Interestingly, while similar CO_2_ assimilation rates were observed in both species under control conditions, Gs values in *O. coarctata* were only half of *O. sativa* (figure 2a,b), resulting in a 73% higher intrinsic WUE (iWUE) in the leaf of *O. coarctata* (figure 2e).

**Table 1.** Parameters derived from CO_2_ and light response curves (*A/Ci* and *A/I* curves, respectively). Parameters calculated from *A/Ci* curve: *V*_cmax_ (μmol (photons) m^−2^ s^−1^)— maximum carboxylation rate of RuBisCO, *J*_max_ (μmol (photons) m^−2^ s^−1^)—maximum rate of RuBP regeneration via electron transport. Parameters calculated from *A/I* curve: *I*_comp_ (μmol (photons) m^−2^ s^−1^)—light compensation point, *I*_max_ (μmol (photons) m^−2^ s^−1^)—the point at which Pn does not change significantly as *I* increase, *P*_*N(*Imax*)*_ (μmol (CO_2_) m^−2^ s^−1^)—net photosynthetic rate when *I* = *I*_max_, Փ _(Icomp-I200)_ (μmol (CO_2_) m^−2^ s^−1^)—maximum quantum yield of the assumed linear part (from *I*_comp_ to 200 μmol photon m^–2^ s^–1^) of the light curve. C—cultivated rice, W—wild rice, control—0 mM NaCl, salt—100 mM NaCl treatment. Values are mean ± SE (*n* = 3–4). Different uppercase letters indicate significant differences with *p* < 0.05 by ANOVA.

	calculated variables	C-control	C-salt	W-control	W-salt
*A/Ci* curve	*V*_cmax_	85.2 ± 5.8 A	41.8 ± 2.5 C	58.9 ± 3.8 B	78.0 ± 1.3 A
	*J*_max_	144.3 ± 10.4 A	83.2 ± 2.4 C	102.2 ± 2.8 B	130.5 ± 0.5 A
*A/I* curve	*I*_comp_	8.4 ± 1.8 A	10.7 ± 2.4 A	16.0 ± 3.4 A	11.9 ± 0.8 A
	*I*_max_	468.3 ± 34 AB	205.3 ± 50.5 C	397.7 ± 35.4 B	542.3 ± 58.8 A
	*P*_*N*(Imax)_	8.5 ± 0.9 A	3.2 ± 1.1 B	7.8 ± 0.6 A	9.9 ± 0.9 A
	*Փ*_(Icomp-I200)_	0.029 ± 0.002 A	0.015 ± 0.004 B	0.031 ± 0.001 A	0.032 ± 0.001 A

The analysis of CO_2_ and light response curves showed that salt-treated cultivated rice had lower Pn than the controls while it was not the case in wild rice (figure 2f,g). The lower maximum rate of RuBisCO carboxylation (*V*_cmax_) is one of the factors that restrict the CO_2_ assimilation rate. *V*_cmax_ reduced significantly (by 51%) in *O. sativa* but increased by 32% in *O. coarctata* after prolonged saline treatment, indicating that the altered RuBisCO carboxylation process is partially responsible for the changes in photosynthetic rate (table 1). The CO_2_ assimilation rate was also limited by the maximum rate of electron transport during the RuBP-regeneration stage (*J*_max_). Supplying NaCl solution instead of water showed a notable impact on *J*_max_ (−43%/+27% in *O. sativa* and *O. coarctata*, respectively; table 1). *V*_cmax_ and *J*_max_ were dramatically affected by salt treatment in both species, implying that the amount or activity of RuBisCO and the synthesis of RuBP in rice species is very sensitive to saline conditions.

In addition, parameters derived from the light curve (*A*/*I* curve) were also influenced by salinity (table 1). From *I*_comp_ to *I*_200_ (*I* = 200 μmol photon m^–2^ s^–1^), an assumed linear response of Pn is commonly used to calculate maximum quantum yield (Φ_(Icomp-I200)_). Φ_(Icomp-I200)_ changes in table 1 indicated that salt-treated seedlings of *O. sativa* could only fix half of the amount of CO_2_ with the same quantity of photons compared to the control, while no difference was found in *O. coarctata*. Also, salinity suppressed Pn_(Imax)_ in cultivated rice; therefore, smaller *I*_max_ was needed, but an opposite scenario was observed in wild rice (table 1).

### (b) Stomatal morphology and operation are unaffected in *Oryza coarctata*

**Figure 3.**
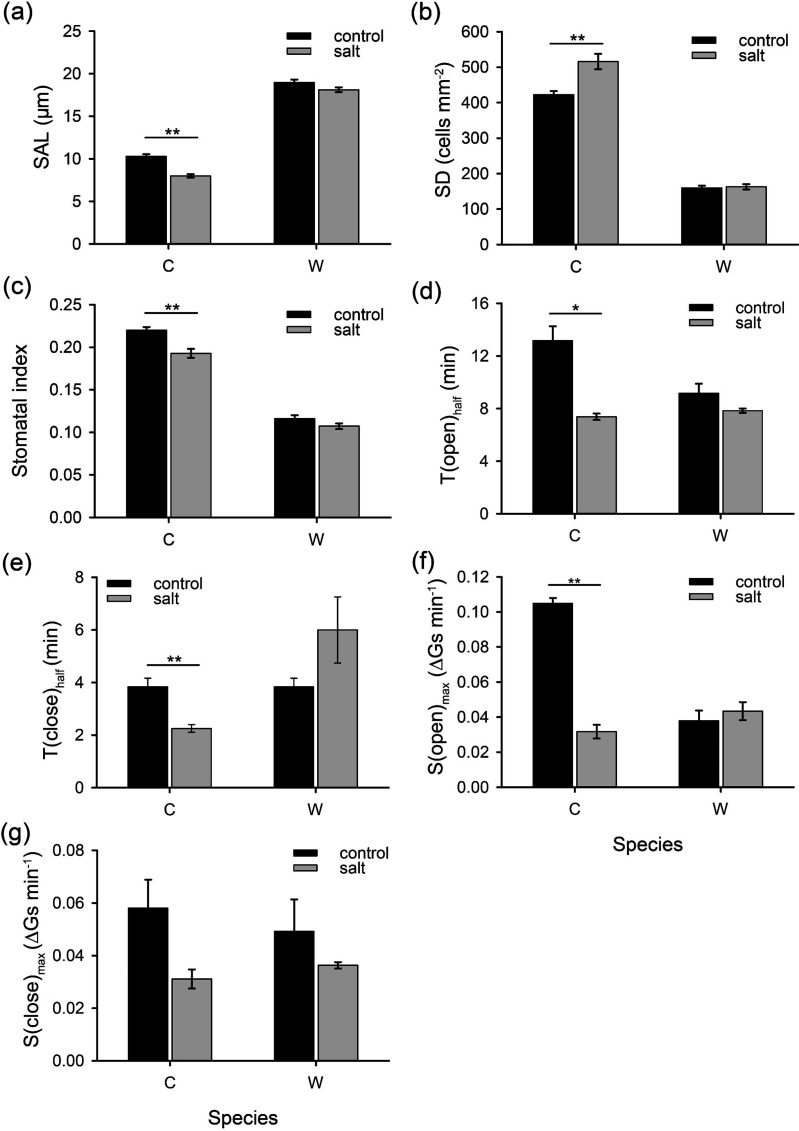
Stomatal development and operation differ between cultivated and wild rice. (a) SAL—stomatal aperture length; (b) SD—stomatal density; (c) SI—stomatal index. Measurements were taken after treating plants with 100 mM NaCl for one month. (d,e) Stomatal operation response to light/dark stimulation: response time to reach half of Gs for opening (d) *T*(open)_half_ and closing (e) *T*(close)_half_; maximum operation speed for opening (f) *S*(open)_max_ and closing (g) *S*(close)_max_. The schematic for the calculation method is shown in figure 1. C—cultivated rice *O. sativa*, W—wild rice *O. coarctata*. Values are mean ± SE ((a) *n* = 21–41, (b,c) *n* = 16–18, (d–g) *n* = 3–4). * and ** indicate significant differences between species with *p* < 0.05 and *p* < 0.01 by Student’s *t*‐test, respectively.

NaCl treatment affected the cell size and density on the leaf surface of *O. sativa*, but no alterations were observed in *O. coarctata* (figure 3 and electronic supplementary material, figure S3). In cultivated rice, the SAL decreased by 23% when grown in saline soil, whereas in wild rice, it remained unchanged (figure 3a). However, the stomatal aperture of *O. coarctata* was significantly larger than *O. sativa* in non-salt and salt conditions (1.8- and 2.3-fold, respectively; figure 3a and electronic supplementary material, figure S3). Moreover, the SD of cultivated rice increased by 22% under salinity conditions (figure 3b); this was most likely due to the leaf surface becoming denser (electronic supplementary material, figure S3). Meanwhile, salt treatment did not cause any impact on stomatal morphology in *O. coarctata* (figure 3a–c). Under all conditions, the SD of halophytic rice was found to be 2.6- to 3.2-fold lower than that of the glycophytic species (figure 3b), which could be associated with the fact that *O. sativa* has a generally higher SD than wild species [5]. The SI in salt-treated seedlings of *O. sativa* was considerably lower than in control (figure 3c), suggesting that cultivated rice may regulate stomatal numbers via developmental mechanisms in response to salinity stress. Such a decrease in SI explains the lower Pn (figure 2a) under salt conditions. On the contrary, the SI in wild rice was not influenced by NaCl (figure 3c).

Rapid stomatal response is crucial for plants to balance water loss and CO_2_ absorption, especially under salinity stress. The half response time to light (stomatal opening) and dark (stomatal closure) in *O. sativa* were significantly shorter after salt treatment (figure 3d,e). This may be attributed to the fact that salinity stress led to lower Gs (figure 2b); thus, it took less time to attain steady-state Gs. As shown in figure 3f, a 70% drop in maximum stomatal opening speed was observed in cultivated rice, implying NaCl had a considerable impact on the ability of stomatal movement. However, the changes of all these parameters including maximum stomatal opening speed in *O. coarctata* were not statistically significant (figure 2).

### (c) Excised leaves of *Oryza coarctata* are less affected by salt treatment

**Figure 4.**
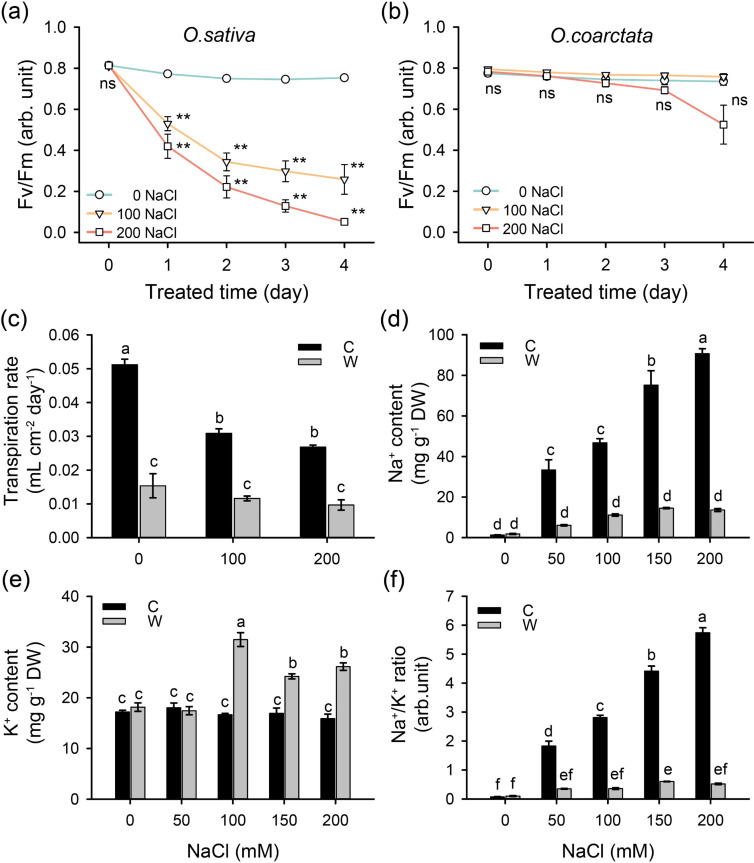
Salinity effects on photochemistry, ion content and transpiration rate of the excised leaves of cultivated and wild rice species. Maximum potential quantum yield of PS II (Fv/Fm) of (a) cultivated and (b) wild rice excised leaves in response to various NaCl (mM) for 4 days (*n* = 6); samples were 30 min dark-adapted before the measurement. (c) Average daily transpiration rate, (d) Na^+^ content (mg g^−1^ dry weight), (e) K^+^ content (mg/g dry weight) and (f) K^+^/Na^+^ ratio of excised leaves treated with various NaCl levels for 3 days. C—cultivated rice *O. sativa*, W—wild rice *O. coarctata*. Values are mean ± SE (*n* = 3–4). ** indicates significant differences between salt treatments (100 and 200 NaCl) and control (0 NaCl) with *p* < 0.01 by Student’s *t*‐test; ns stands for no significant difference. Different letters indicate significant differences with *p* < 0.05 by ANOVA.

As whole-plant responses in Pn/Gs may be influenced by the differential ability of roots to take up/exclude toxic Na^+^, experiments on excised leaves were conducted to ensure that leaf mesophyll cells receive the same salt load in both species. Under salt treatment, Fv/Fm declined dramatically by day 1 (by 32–46%) dropping further to only 7% of control (for 200 mM NaCl treatment) by day 4 (figure 4a). In *O. coarctata*, the maximum quantum yield of PSII was not significantly affected by salinity in the first 3 days (figure 4b). The transpiration rate of excised leaves from cultivated rice was 3.4-fold higher than wild rice under control conditions and decreased by 39.7 and 47.6% relatively to control in response to 100 and 200 mM NaCl, respectively (figure 4c). The respective changes in transpiration in *O. coarctata* were 24.2 and 36.9% for 100 and 200 mM NaCl, respectively (figure 4c). Although there were 2.5- to 2.7-fold differences in Tr between two species under 100 and 200 mM NaCl conditions (figure 4c), the differences in Na^+^ content under the same conditions were 4.2- to 6.7-fold (figure 4d). Thus, the observed stronger inhibition of PSII operation in *O. sativa* cannot be merely explained by the higher Na delivery to leaf mesophyll. Although the K^+^ content in wild rice was higher in 100–200 mM NaCl levels (figure 4e), the Na^+^/K^+^ ratio mainly depended on the changes in Na^+^ content (figure 4f).

### (d) Oryza *coarctata* shows reduced sensitivity to exogenously applied abscisic acid

**Figure 5.**
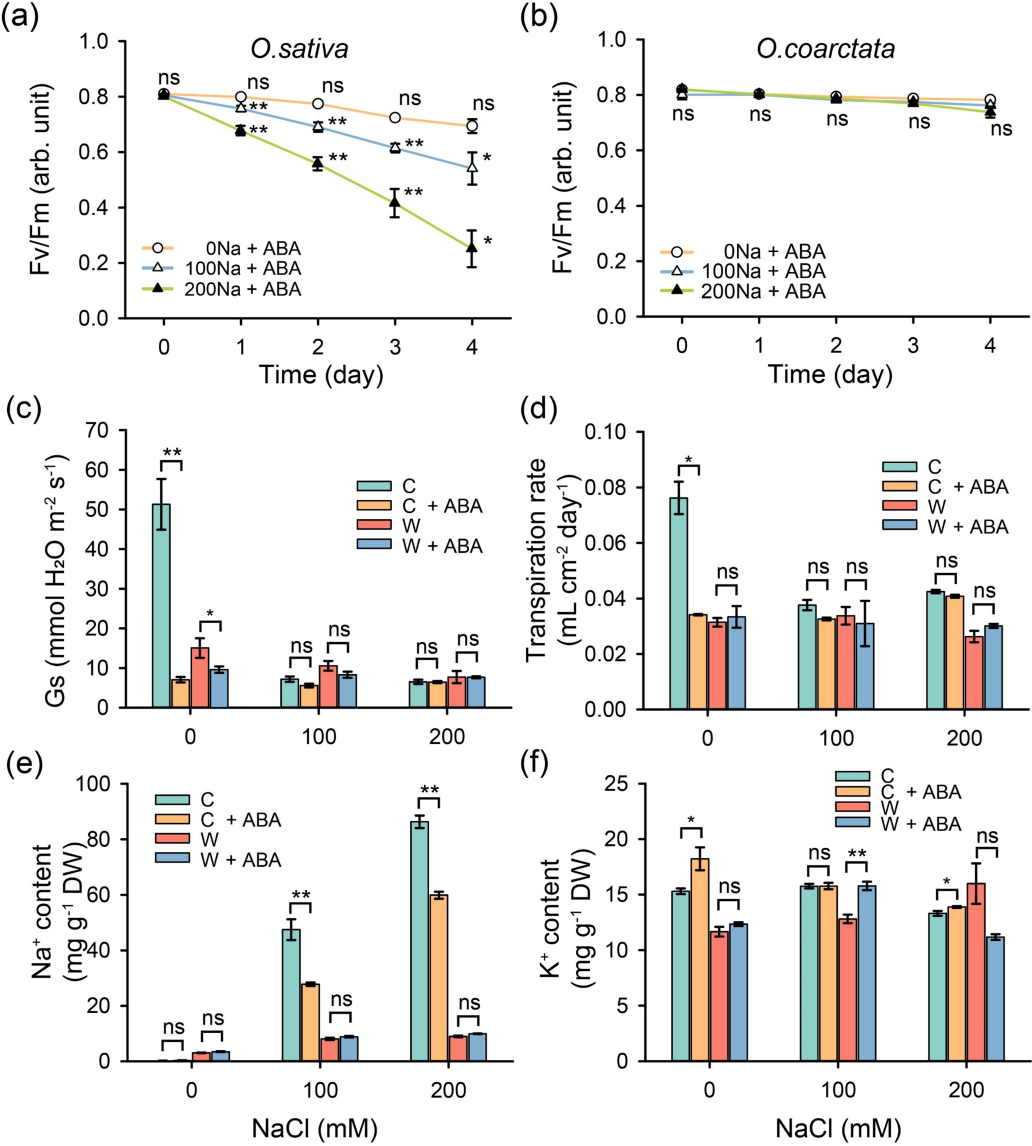
Effects of ABA (100 µM) and NaCl (100 or 200 mM) treatment on photochemistry, stomatal conductance, transpiration rate and ion content of excised leaves. Fv/Fm measured from (a) cultivated and (b) wild rice treated for 4 days, samples were 30 min dark-adapted before the measurement. (c) Stomatal conductance (Gs) after 5 h of treatment. (d) Average daily transpiration rate, (e) Na^+^ and (f) K^+^ content (mg g^−1^ dry weight) for 3-day treatment. C—cultivated rice *O. sativa*, W—wild rice *O. coarctata*. Values are mean ± SE (*n* = 3–6). * and ** indicate significant differences between +ABA and −ABA treatments with *p* < 0.05 and *p* < 0.01 by Student’s *t*‐test, respectively; ns stands for no significant difference.

Ionic relations in mesophyll are ultimately determined by the amount of NaCl accumulated in the leaf lamina, and the latter may be affected by the difference in the transpiration rate. To exclude this uncertainty, experiments on excised leaves treated with ABA were conducted (ensuring that stomata are closed and the transpiration rate is the same between species). In total, 100 µM exogenous ABA alleviated the NaCl-induced damage to PSII in cultivated rice (figures 4a and 5a). In the meantime, the PSII activity was found unaffected in salt-treated *O. coarctata*, and additional ABA treatment did not affect Fv/Fm ratio (figures 4b and 5b). After supplying the ABA, Gs and Tr declined by 87 and 56% in *O. sativa* under control conditions (figure 5c–d). However, under salt conditions, both Gs and Tr of cultivated rice were relatively low, regardless of the addition of ABA. Furthermore, supplying ABA considerably reduced the leaf Na^+^ content in cultivated rice under salt conditions (figure 5e), suggesting that ABA alleviates PSII operation by regulating the Na^+^ delivery to the leaf lamina. At the same time, halophytic rice were insensitive to ABA treatment, as none of the parameters were affected by exogenous ABA (figure 5).

Surprisingly, despite both species having similar Tr for NaCl + ABA treatments (figure 5d), the Na^+^ content in cultivated rice leaves was 2.2- to 5.1-fold higher than that in halophytic species (figure 5e), indicating that *O. coarctata* also used other strategies (e.g. salt secretion) to maintain a relative low Na^+^ content in excised leaves. No clear trends were observed for K^+^ content, (figure 5f).

### (e) Potential for utilizing Na^+^ for photosystem II and stomatal operation in *Oryza coarctata*

**Figure 6.**
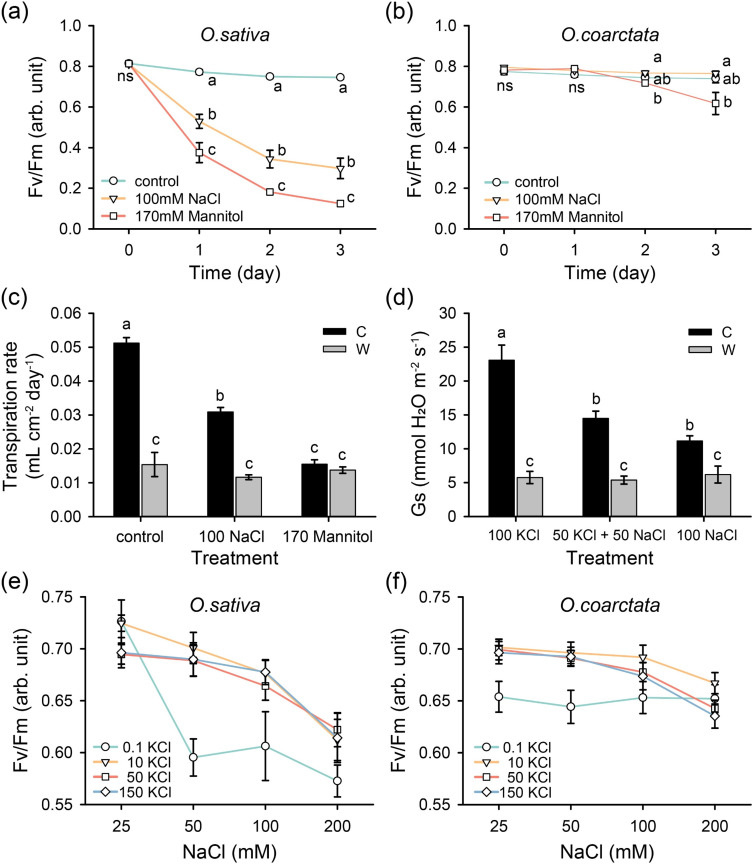
Isotonic mannitol and K/Na substitution treatments on excised leaves and leaf segments. (a–c) NaCl or isotonic mannitol and (d) various K/Na treatments on excised leaves for 3 days (*n* = 3–9). Fv/Fm of (e) cultivated and (f) wild rice leaf segments floated on various K/Na solutions (mM) for 3 days (*n* = 15–16); samples were 30 min dark-adapted before the measurement. C—cultivated rice *O. sativa*, W—wild rice *O. coarctata*. Values are mean ± SE. Different letters indicate significant differences with *p* < 0.05 by ANOVA. ns stands for no significant difference.

Salinity stress may affect the operation of PS machinery by both imposing ion toxicity (ion effect) or reducing water availability (osmotic component of salt stress). To distinguish between these two possibilities, 170 mM mannitol treatment (isotonic to 100 mM NaCl) was applied to excised leaves and compared with NaCl treatment. Compared to NaCl treatment, osmotic treatment induced lower Fv/Fm and Tr in *O. sativa* but not in *O. coarctata* (figure 6a–c). This suggests that both rice species may use NaCl for cellular osmotic adjustment. However, the substitution of K^+^ by Na^+^ reduced Gs in cultivated rice but not in wild rice (figure 6d), indicating that K^+^ is more crucial for the stomatal operation in *O. sativa*, while wild rice is capable of utilizing Na^+^ for stomatal operation.

To determine whether the availability of Na^+^ and K^+^ also impacts the PSII operation in rice species, the floating leaf segments method was used to exclude any possible effects contributed by transpiration. Increasing salinity levels resulted in a progressive decline in Fv/Fm values in cultivated rice (figure 6e). In the presence of low K^+^ (0.1 mM), the Fv/Fm of *O. sativa* dropped considerably under 50 mM Na^+^ concentration (figure 6e). On the contrary, Fv/Fm values in wild *O. coarctata* were stable and not affected by increasing NaCl levels (figure 6f). Overall, these results are suggestive of PSII in *O. coarctata* being able to operate efficiently even in the absence of available K^+^.

## 4. Discussion

### (a) The stomatal patterning was unaffected in *Oryza coarctata* under saline conditions

Under control conditions, Gs of wild rice was about half of the cultivated rice (figure 2b). However, Pn was only 15% less than that in cultivated species (figure 2a), which represents a much higher iWUE in *O. coarctata* (figure 2e). Low Gs, which also leads to low Tr, was most likely due to low SD on the abaxial surface (figure 3b) [44]. The difference in SD and size between the two species can be explained by different leaf structures. Unlike wild *Oryza* species, cultivated species possess smaller but numerous stomata and higher Gs, leading to low WUE [5]. While cultivated rice species close their stomata when exposed to salinity, higher stomata density implies a much higher proportion of (non-productive) residual transpiration, causing unnecessary water losses and further compromising plant performance. Under stress conditions, residual transpiration may contribute up to 50% of total water loss by plants during the day and 60% during the night [45]. Also, the high iWUE in wild rice indicated less water use for CO_2_ assimilation, which may contribute to less salt uptake under salinity conditions.

Regulation of stomatal formation in plants is complex and related to many transcriptional factors (TF) [17,46]. In cultivated rice, *OsSPCH* (*SPEECHLESS*), *OsICE* (*INDUCER OF CBF EXPRESSION*) and *OsMUTE* are deemed essential for the development of stomata, controlling the initiation of the stomatal lineage, guard mother cell transition and formation of subsidiary cells [47]. It remains to be answered whether, under both control and salinity conditions, similar TFs contribute to the special stomatal pattern in *O. coarctata*, or if there is any unique pathway(s) that regulates stomatal development in a wild rice relative.

After being treated with NaCl for a month, the SD of new leaves increased in *O. sativa* because the leaf became smaller and thicker, while the Gs decreased considerably due to the reduction in SAL and SI (figure 3), which then led to less diffusion of CO_2_ in mesophyll and eventually the decrease in Pn (figure 2). However, the salt stress did not affect the stomatal formation and photosynthesis in *O. coarctata*, as thick leaves and cuticle layers may help to preserve water, and large stomata ensure CO_2_ uptake. Notably, in *O. sativa* species, some rice varieties with intrinsic low SD and large stomata performed less well in response to salinity treatment than those with high SD and small stomata, and their stress-induced stomatal closure speed is different [11]. Hence, in addition to the patterning, stomatal operation is also vital for stress tolerance.

### (b) Salt reduced the speed of stomatal movement in cultivated but not in wild rice

Stomatal operation relies on the changes in the turgor and volume of GCs, which are regulated by rapid transport of inorganic ions and biosynthesis of organic compounds such as malate [15,17]. Faster stomatal opening and closure imply better WUE and less yield penalty [12], and the speed of Gs response can be affected by both anatomical (e.g. SD and size) and biochemical factors (e.g. density and activity of transporters) [16]. In this study, the half response time of stomatal opening and closing was considerably less in salt-treated *O. sativa* compared to control (figure 3d,e). From an anatomical perspective, this is mainly because salt-induced smaller stomata have lower Gs (figure 2b); thus, less response time is needed in species with dumbbell-shaped GCs [4]. However, when focusing on the stomatal maximum opening/closing speed in figure 3f,g, other factors, such as the operation of transporters and ion channels, become predominant.

K^+^ and Cl^−^ accumulations in GCs are crucial for cell swelling and a subsequent stomatal opening [16]. *Oryza sativa* has a significantly higher leaf Na^+^ content than *O. coarctata* after long-term salt treatment [48]. Stomatal movements of salt-treated cultivated rice were considerably slower than control plants, but no such pattern was found in wild rice (figure 3f) in the present study. It may be suggested that Na^+^ accumulation blocks inward KAT/AKT and outward GORK potassium channels in GCs [15], thus affecting K^+^ transmembrane transport and, consequently, stomatal movements. On the other hand, the Gs of excised leaves from wild rice were not affected by the substitution of K^+^ with Na^+^ (figure 6d), suggesting that *O. coarctata*, as a halophyte, is capable of utilizing the non-toxic level Na^+^ as a cheap source for osmoregulation, which assures the unaffected stomatal operation under saline conditions [15,49]. Hence, the fast stomatal kinetics after salt treatment could be considered as a beneficial trait to be targeted in breeding programmes, making the plants perform better in saline soil.

### (c) Maximum rates of carboxylation of ribulose bisphosphate carboxylase and electron transport through photosystem II for regeneration of ribulose 1,5-bisphosphate were higher in wild rice

In this study, both *V*_cmax_ and *J*_max_ were suppressed by salt in *O. sativa*, while the opposite scenario occurred in *O. coarctata* (table 1). The large subunit of RuBisCO, RbcL, is localized in chloroplasts [50]. Previous proteomic analysis showed that salinity treatment led to a threefold increase in the amount of RuBisCO large subunit as well as RuBisCO activase in *O. coarctata* but not in *O. sativa* [25]. This may explain our finding that salt treatment stimulated *V*_cmax_ in *O. coarctata* (table 1) because of the increase in RuBisCO amount and activity.

Chloroplastic FBPase is known as a regulatory enzyme in the Calvin cycle that can increase RuBisCO activity and RuBP content [51]. Despite that both biochemical and immunological properties of chloroplastic FBPase are the same in *O. sativa* and *O. coarctata*, the isolated chloroplastic FBPase from *O. coarctata* exhibits higher salt tolerance than the chloroplastic FBPase isolated from *O. sativa* [36]. Also, when the chloroplastic FBPase-coded gene (*PcCFR*) from *O. coarctata* is introduced to cultivated rice, the transgenic plant shows enhanced salt tolerance [52]. Combined with the current results (table 1), salt-tolerant chloroplastic FBPase in wild rice could be one of the reasons for its high RuBisCO activity and RuBP-regeneration rate under saline conditions.

Chloroplastic fructose-1,6-bisphosphate aldolase (ALDP) is a key enzyme that controls the conversion of fructose-1,6-bisphosphate and G3P in glycolysis and gluconeogenesis, and in *O. sativa* chloroplast, aldolase is sensitive to ROS stress [53]. As salinity stress triggers ROS accumulation in the leaf, the susceptibility of chloroplast aldolase in cultivated rice may be another reason for salt-induced low efficiency in CO_2_ assimilation.

### (d) Photosystem II in wild rice performed better than in cultivated rice under salt conditions

In addition to the CO_2_-fixing enzymes, the dysfunction of the PSII is another factor that limits photosynthesis under saline conditions. In this study, the disintegration of PS caused *I*_*max*_ to considerably decrease in salt-treated cultivated rice, as the decline in Φ_(Icomp-I200)_ indicates that the PS of *O. sativa* had lower efficiency in utilizing photons to fix CO_2_ (table 1). Moreover, the Fv/Fm in both excised leaves and floating leaf segment experiments were highly sensitive to salt treatment, indicating the susceptibility of PSII in *O. sativa* (figures 4 and 6). This was not the case for *O. coarctata*. The decreased Fv/Fm is predominantly a consequence of inactivation or damage of the PSII reaction centres. It was shown that in *O. coarctata*, salt treatment induced several chloroplast proteins, which are related to light capture in PSs, stabilization of the PSII reaction centre and Calvin cycle [25]. These results demonstrated that the PSs in *O. coarctata* have greater salt tolerance than that in *O. sativa*.

Chloroplast ultrastructure alteration caused by salt-induced ROS accumulation and ionic imbalance can also impact PS activity [24]. With the presence of NaCl, low K^+^ supply drastically reduced Fv/Fm in cultivated rice but not in *O. coarctata* (figure 6), implying that potassium is more crucial for *O. sativa* and salt may be vital for PSII activity in halophytic rice. The expression of genes related to K^+^ retention (high-affinity K^+^ gene (*HAK1*), high-affinity potassium transporter gene (*HKT1*) and salt-overly-sensitive gene (*SOS1*)), H^+^-ATPase (*AHA1*) and Na^+^ sequestration (Na^+^/H^+^ exchanger gene (*NHX1*) and vacuolar-type H^+^-ATPase subunit c gene (*VHA-c*)) in *O. coarctata* leaf are significantly upregulated under salt treatment compared to cultivated rice [48,54], which is consistent with this study that *O. coarctata* maintained optimal PSII activity under salt conditions due to better utilization of K^+^, sufficient energy supply and efficient Na^+^ vacuolar sequestration.

### (e) Leaves of wild rice were insensitive to exogenous abscisic acid

Exogenous ABA application caused stomatal closure and reduced Na^+^ accumulation, therefore alleviating salt impact on the PSII activity of *O. sativa* (figure 5). Interestingly, *O. coarctata* showed ABA insensitivity in Gs, PS activity and ion content under both control and salt conditions (figure 5), which may relate to regulation in the ABA sensing pathway and protectants synthesis. In halophyte *Thellungiella*, under salt conditions, ABA receptor genes (pyrabactin resistance 1-like (*PYL*) genes *PYL1*, *PYL4*, *PYL5* and *PYL6*) and S-type anion channel gene (*SLAC1*) in GCs are strongly suppressed, therefore maintaining stable stomatal conductance; on the contrary, *Arabidopsis*, with Gs decline under salt conditions, has the whole ABA signalling pathway upregulated in GCs [55]. A gene *Multiprotein bridging factor 1* a (*MBF1a*) from halophyte *Hordeum brevisubulatum* showed the capability of enhancing salt tolerance and ABA insensitivity in the *Arabidopsis* overexpression line [56], and salt stress-induced ABA elevation was about fourfold lower in halophytes compared with glycophytic crops [15]. The physiological implication of these findings and the mechanism basis of ABA-insensitive in *O. coarctata* are the subjects of future studies. A recent bioinformatic analysis using sequence alignment of PYR1 from different species revealed that the probability of the amino acid residue near the gate loop mutating from S (serine) to A (alanine) increases in drought-tolerant species [57], and targeted activation mutations of amino acid residues in PYR1 enhanced the basal level of interaction between PYR1 and type 2C protein phosphatase (PP2C) [58]. This prompted a suggestion that in stress-tolerant species, both the increased number of PP2C copies and the amino acid residue mutations in PYR1 could be two of the mechanisms by which plants have evolved to respond to drought stress by altering their ABA signalling sensitivity. It remains to be determined whether this is also true for *O. coarctata*, and whether these traits can then be introduced into cultivated *O. sativa* species.

## 5. Conclusion

Compared to salt-sensitive cultivated *O. sativa*, wild rice, *O. coarctata,* performed much better when exposed to saline conditions. This difference was attributed to improved leaf photochemistry and leaf gas exchange characteristics, including unaltered stomatal-related traits (both developmental and operational), enhanced RuBisCO carboxylation and RuBP regeneration in the Calvin cycle, more stable PS activities and efficient photons utilization. *Oryza coarctata* is also capable of efficiently replacing K^+^ by Na^+^ for stomata operation. These traits can be potentially targeted in the breeding programme for salt-tolerance enhancement in cultivated rice, especially in the light of the fact that a high-quality chromosome-level genome sequence of *O. coarctata* has been recently released [59].

## Data Availability

Supplementary figures are provided in the electronic supplementary material [60].
