# Correction: ‘Learning from nature: photosynthetic traits conferring superior salt tolerance in wild rice *Oryza coarctata*’ (2025), by Ping *et al.*

**DOI:** 10.1098/rstb.2025.0235

**Published:** 2025-11-06

**Authors:** Ping Yun, Babar Shahzad, Md Hasanuzzaman, Tahmina Islam, Lana Shabala, Meixue Zhou, Gayatri Venkataraman, Zhong-Hua Chen, Sergey Shabala

**Affiliations:** ^1^ The University of Western Australia, Perth, Australia; ^2^ University of Tasmania, Hobart, Tasmania, Australia; ^3^ Sher-e-Bangla Agricultural University, Dhaka, Dhaka District, Bangladesh; ^4^ Department of Botany, University of Dhaka, Dhaka, Bangladesh; ^5^ MS Swaminathan Research Foundation, Chennai, Tamil Nadu, India; ^6^ School of Agriculture, Food and Wine, Waite Research Institute, University of Adelaide, Adelaide, Australia

**Keywords:** wild rice, salinity, photosynthetic limitations, stomatal operation, non-stomatal limitations, ABA insensitive, gas exchange

*Phil. Trans. R. Soc. B* **380**: 20240242 (Published online 29 May 2025). (https://doi.org/10.1098/rstb.2024.0242)

An author’s affiliation was incorrectly given in the original publication. Tahmina Islam’s affiliation has now been corrected to ‘Department of Botany, University of Dhaka, Dhaka, Bangladesh’.

This has been corrected on the publisher's website.

